# A short review of the electrochemical technologies for pit arrays fabricated on the surfaces of indium phosphide wafer

**DOI:** 10.1016/j.heliyon.2023.e16800

**Published:** 2023-05-29

**Authors:** Xiaobin Lu

**Affiliations:** School of Chemistry and Chemical Engineering, Qiannan Normal University for Nationalities, Duyun, 558000, Guizhou, China

**Keywords:** Indium phosphide, Pit array, Electrochemical method, Fabrication, Anodization

## Abstract

Fabricating a pit array on the surface of indium phosphide wafer can change its photoelectric properties, improve its photoelectric conversion efficiency, and expand its application range. There are few reviews devoted to the fabrication of regular hole arrays on the surface of indium phosphide wafers by electrochemical methods. In this paper, twelve electrochemical approaches for assembling pit arrays on the surface of indium phosphide wafers were introduced, the structure and experimental process of the electrochemical device were highlighted, and the resulting top and section views were also shown by animation. It can provide a useful reference guide for the large-scale fabrication of regular hole arrays on the surface of indium phosphide wafers.

## 1 Introduction

Among many semiconductor materials, indium phosphide (InP) has been considered as one of the most promising photoactive material for solar energy conversion due to its smaller direct band gap [1], suitable band edge location and low carrier recombination rates. However, current researches on InP-based photoanodes are mainly based on planar structures.

For planar structures, a large amount of incident light is reflected away from the surface, limiting the solar energy conversion efficiency. Therefore, in order to improve the light conversion efficiency of solar cells, it is necessary to reduce the light reflectivity on the surface of these devices. In this regard, one-dimension nanostructures such as nanowires and nanotubes have advantages because they can offer some properties that are superior to that of planar structures. First, one-dimension nanostructure arrays can enhance light absorption by reducing the reflection of incident light and increasing the length of photon path [2,3]. Second, the one-dimension nanostructure array can decouple the light absorption and the carrier collection direction, and provide a unidirectional conductive channel that enhances electron-hole separation and charge transport, thereby reducing the recombination of electron-hole pairs [[4], [5], [6], [7], [8]]. In addition, one-dimension nanostructures have a larger surface area [9], which can accelerate charge transfer and electrochemical reactions, reduce the overpotential required for oxidation reactions, and enhance chemical stability against corrosion [10].

High-density nanopore arrays are one of the most promising nanostructures among various structures due to their large surface area and low light reflectivity [[11], [12], [13]]. Porous structure materials have great potential future application in photoelectric conversion devices [14,15], such as solar cells [[16], [17], [18], [19]], photoelectrochemical hydrogen production [[20], [21], [22]], and photodetectors [16,[23], [24], [25], [26], [27], [28]].

Among all the nanopore fabrication methods, the electrochemical etching has been the most commonly used because of its simple experiment set-up and the controllable prepared nanopore morphology [29]. Many nanoporous materials have been assembled by this method, such as GaAs [13,30,31], Si [17,18,26,32], GaP [23,28,[33], [34], [35], [36]] and GaN [13,37,38]. Electrochemical etching and applications of InP nanopores have also attracted a lot of attention [27,[39], [40], [41], [42], [43], [44], [45]]. However, there are few specific reviews on electrochemical etching experimental devices and methods for InP. Therefore, in this paper, twelve electrochemical methods for fabricating hole arrays on the surface of InP wafers were introduced, and the structure and experimental process of the electrochemical set-up were highlighted [32,[42], [43], [44],[46], [47], [48], [49], [50], [51]]. It provided a useful reference guide for the large-scale assembly of regular hole arrays on the surface of InP sheets.

## 2 Electrochemical etching equipments and processes for InP wafers

Many methods for the self-fabrication of nanopit arrays have been realized, among which electrochemical etching has found to be a better tool for the formation of two-dimensional nanostructures on the surfaces of InP wafers. According to prior studies, the electrochemical etching condition has a key effect on pit formation and morphology in porous InP, such as electrolyte types and concentration, current density, voltage, doping type, crystalline directions, temperature etc. 12 typical methods of electrochemical etching for the fabrication of nanopore pit array on the surfaces of InP were chosen to illustrate their process diagram, experimental details and resulting morphology.

### 2.1 Electrochemical etching method 1

**Fig. 1:**
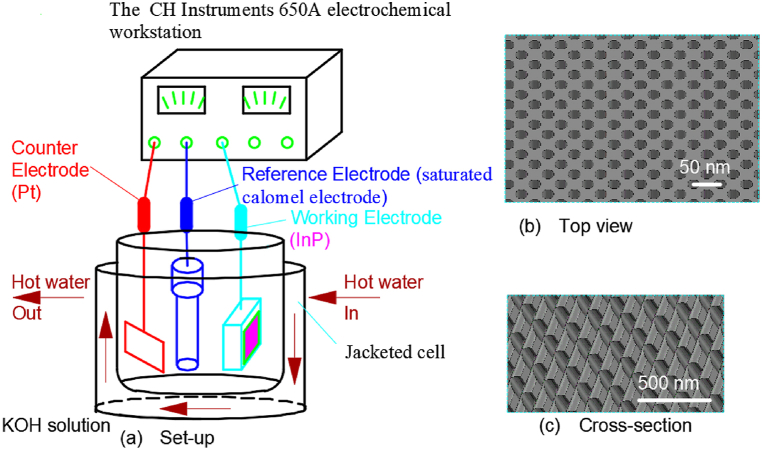
The set-up for anodic formation of nanoporous InP in KOH electrolytes.

Nathan Quill et al. investigated the effects of temperature and concentration on InP nanopit formed by anodization in a KOH solution [52]. The whole experimental set-up was illustrated in Fig. 1(a). A platinum counter-electrode, a saturated calomel reference electrode, and InP working electrode were utilized in the experiment. Temperature control at 10–50 °C was achieved through hot water circulation. The CH Instruments 650 A electrochemical workstation was used for process parameter control and data acquisition. Anodic oxidation was carried out in a potassium hydroxide solution with concentrations from 2.5 mol L^−1^ to 17 mol L^−1^ without light assistance by a linear potential sweep at 2.5 mV s^−1^ with voltages from 0 V to 0.75 V. And the resulting morphology of the pit arrays in Fig. 1(b and c) shows that the pore width is 10–40 nm and the pore depth is 0.8–2.8 μm.

### 2.2 Electrochemical etching method 2

**Fig. 2:**
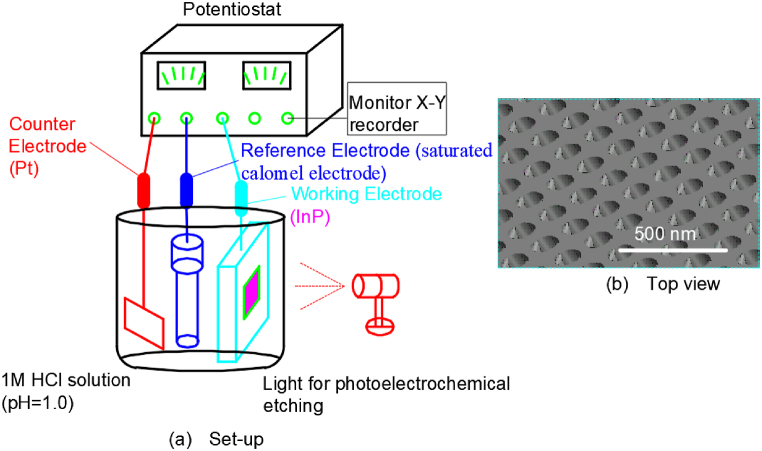
Set-up for electrochemical etching of *n*-InP in a dilute HCl solution.

Chinami Kaneshiro et al. carried out the electrochemical etching on the *n*-InP surfaces, in which the etching parameters were studied and optimized in a dilute hydrochloric acid solution [14]. The whole experimental set-up was presented in Fig. 2(a). To improve electrical conductivity, germanium, gold, and nickel were deposited on the back of the wafer, respectively and annealed. There were an InP working electrode, a platinum counter electrode and a reference saturated calomel electrode in the experiment. The carrier density of n-type InP ⟨100⟩ wafers was 5 × 10^15^ cm^−3^ and a dilute solution of HCl was applied for the electrolyte. Electrochemical etching on the surface of indium phosphide wafer was achieved by anodic reaction. In this study, certain intensity light irradiation was used to induce anodic reaction of indium phosphide wafer. The voltage on the indium phosphide electrode was regulated by a voltage regulator. The etching was initiated using DC pulse voltages from 0 V to 5 V by voltammetry and electrochemical reaction characteristics were studied. And the resulting morphology of the pit arrays in Fig. 2(b) shows that the pit width is 100–300 nm and the pit depth is 0–150 nm.

### 2.3 Electrochemical etching method 3

**Fig. 3:**
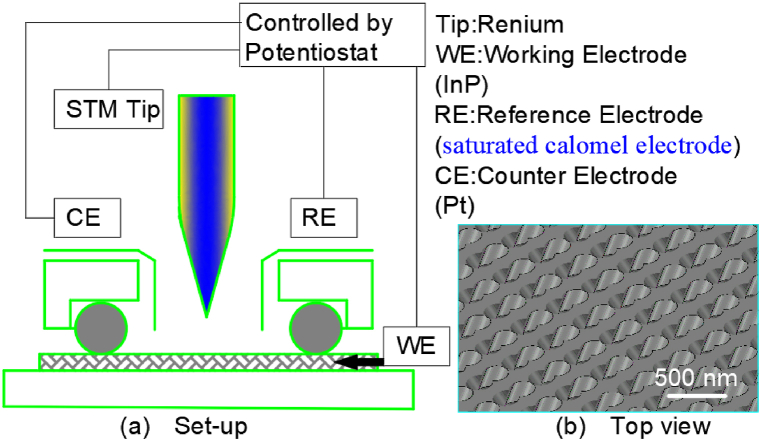
Set-up of in situ electrochemical process for fabrication of porous InP.

Kaneshiro Chinami et al. [15] performed electrochemical experiments in a cell with three electrodes as shown in Fig. 3. The whole experimental set-up was displayed in Fig. 3(a). The carrier content of n-type InP ⟨100⟩ bulk materials was 5 × 10^15^ ∼ l × 10^18^ cm^−3^. In order to increase electrical conductivity, germanium, gold and nickel were deposited on the back of the wafer in sequence and annealed. Before the experiment, the wafers were cleaned with acetone and deionized water. The wafer was then pasted with silver paste to the center of the round copper sheet, and the exposed copper sheet was covered with insulating paint. In a Teflon tank, the copper plate was served as the anode and graphite as the cathode. The surface of indium phosphide wafer was polished with an oxalic acid solution.

Then anodic etching was performed in a dilute hydrochloric acid solution. The sample was then placed in a mixture of hydrochloric and phosphoric acid to remove the irregular layer on the top surface. After ultrasonic cleaning in deionized water for 3 min, a uniform square hole array on the surface of indium phosphide was fabricated.

This experimental device could be used for voltammetry or in situ STM measurement. The DC pulse voltages from 0 V to 2 V on the indium phosphide substrate could be adjusted by the potentiostat. And the resulting morphology of the pit arrays in Fig. 3(b) shows that the pit width is 110–350 nm and the pit depth is 0–10 nm.

### 2.4 Electrochemical etching method 4

**Fig. 4:**
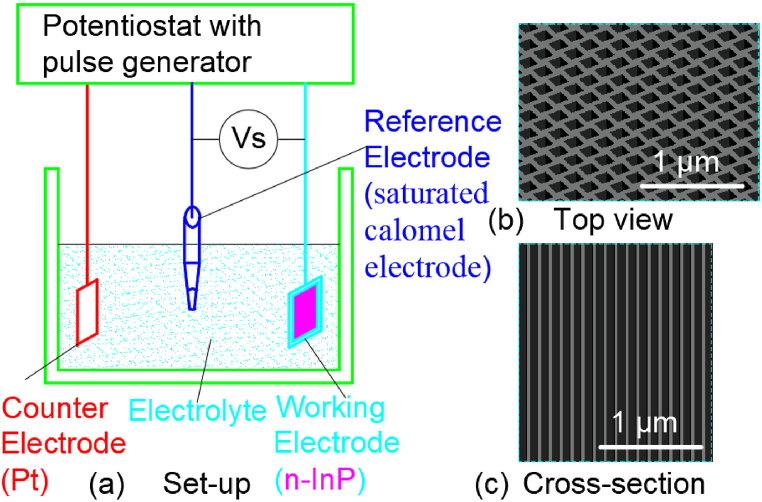
Set-up of the electrochemical process for nanopore formation on InP surface.

Hirano Tetsuro [44] et al. investigated the electrochemical anodization procedure applied for the fabrication of nanopore arrays. The whole experimental set-up was illustrated in Fig. 4(a). An *n*-InP working electrode (n = 1.5 × 10^18^ cm^−3^), a reference saturated calomel electrode (SCE) and a platinum counter electrode were applied in the experiment. The over-potential (Vs) on the InP electrode was manipulated by a potenstiostat. Three electrolytes were used, namely, 1 mol L^−1^ hydrochloric acid (200 mL), a mixture of 1 mol L^−1^ hydrochloric acid (200 mL) and chloroplatin acid (1 g), a mixture of 1 mol L^−1^ hydrochloric acid (200 mL) and nitric acid (3 mL). In order to improve electrical conductivity, germanium, gold and nickel ohmic contact were deposited on the back of the *n*-InP wafer in sequence and annealed. The surface of the wafer was treated with a piranha solution just before the anodic reaction. In this study, DC pulsed voltages from 4 V to 10 V was used to fabricate nanopore arrays on the surfaces of indium phosphide wafers. And the resulting morphology of the pit arrays in Fig. 4(b and c) shows that the pit width is 50–180 nm and the pit depth is 1–23 μm.

### 2.5 Electrochemical etching method 5

**Fig. 5:**
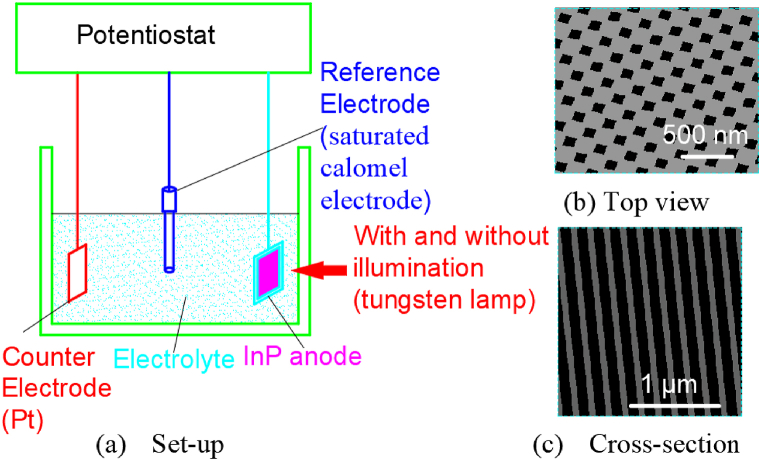
Set-up for electrochemical anodization.

Hajime Fujikura et al. [40] studied electrochemical fabrication of uniform and straight nanopit arrays on the InP Surfaces. The whole experimental set-up was illustrated in Fig. 5(a). An *n*-InP working electrode (n = 1 × 10^18^ cm^−3^), a platinum counter electrode and a reference saturated calomel electrode were used in the experiment. The voltages from 5 V to 9 V on the InP electrode was adjusted by a potentiostat. In this study, two electrolyte solutions (a mixture of 1 mol L^−1^ hydrochloric acid (200 mL), chloroplatin acid (1 g) and ammonium hydroxide (pH = 1) and a mixture of 1 mol L^−1^ hydrochloric acid (200 mL) and nitric acid (3 mL), were used, with or without light irradiation. In order to improve electrical conductivity, germanium, gold and nickel ohmic contact were deposited on the back of the *n*-InP wafer through a traditional metal evaporation in sequence and annealed. The voltages from 5 V to 9 V and anodic oxidation time from 0.5 min to 1 min could be adjusted during the experiment. And the resulting morphology of the pit arrays in Fig. 5(b and c) shows that the pit width is 50–210 nm and the pit depth is 2–80 μm.

### 2.6 Electrochemical etching method 6

**Fig. 6:**
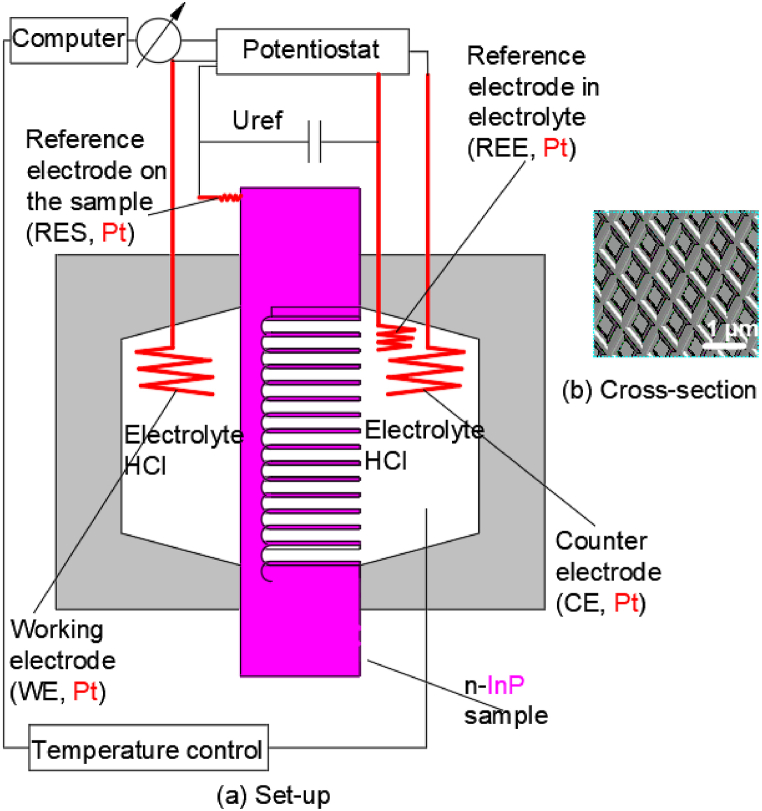
Setup of the electrochemical double cell with four electrodes.

Langa S. et al. [51] performed the anodization etching on the InP surfaces in an electrochemical double cell. The whole experimental set-up was illustrated in Fig. 6(a). The n-type indium phosphide wafer used in this study was doped with silicon with a direction of 100 and the concentration of free electrons in bulk material of 3 × 10^17^ cm^−3^. A Pt reference electrode in the electrolyte (REE), a Pt reference electrode on the sample (RES), a Pt counter electrode (CE), and a Pt working electrode (WE) were utilized in the experiment. The electrodes were connected to a Keithley 236 data analysis equipment. The temperature of the electrolyte was kept constant at 23 °C by a Julabo F25 thermostat only on the surface where the hole grew in the double electrolytic cell. And the resulting morphology of the pit arrays in Fig. 6(b) shows that the pit width is 100–200 nm and the pit depth is 2–15 μm.

### 2.7 Electrochemical etching method 7

**Fig. 7:**
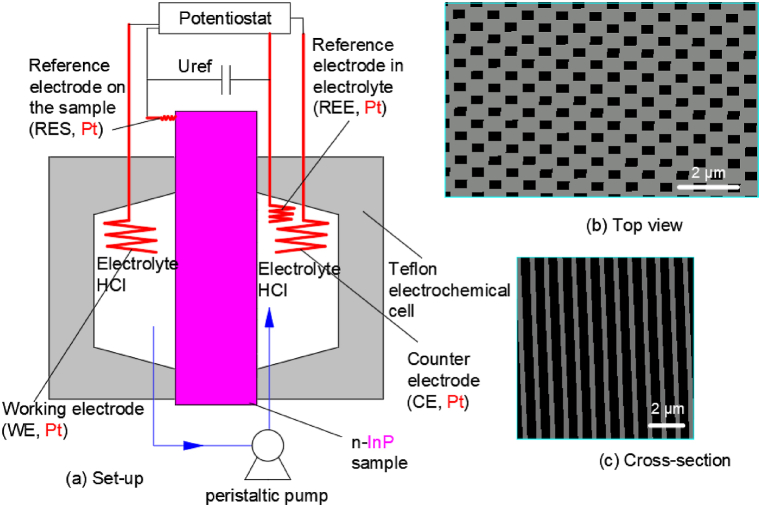
Configuration of the electrochemical double cell using four electrodes with a recycling pump.

Langa S. et al. [11] carried out the electrochemical etching of an InP wafer in a Teflon double cell with four electrodes. The whole experimental set-up was illustrated in Fig. 7(a). Using dual electrolytic cells for good backside contact, the conventional ohmic contact of the indium-gallium/sample was replaced by the electrolyte/sample contact. The currents from 10 mA to 35 mA and voltages from 5 V to 10 V were adjusted by the regulator, and the electrolyte temperature was controlled at 20 °C by the Julabo F25 thermostat. Both the regulator and the thermostat were adjusted by a computer. A recycling pump was used to circulate the electrolyte through the electrolytic cell. The doped concentration of indium phosphide sample was 1 × 10^18^ cm^−3^, and the crystal directions were ⟨100⟩ and ⟨111⟩. The area of the wafer in contact with the electrolyte was 2 square millimeters. Different concentrations of hydrochloric acid were used in this experiment. And the resulting morphology of the pit arrays in Fig. 7(b and c) shows that the pit width is 100–400 nm and the pit depth is 5–50 μm.

### 2.8 Electrochemical etching method 8

**Fig. 8:**
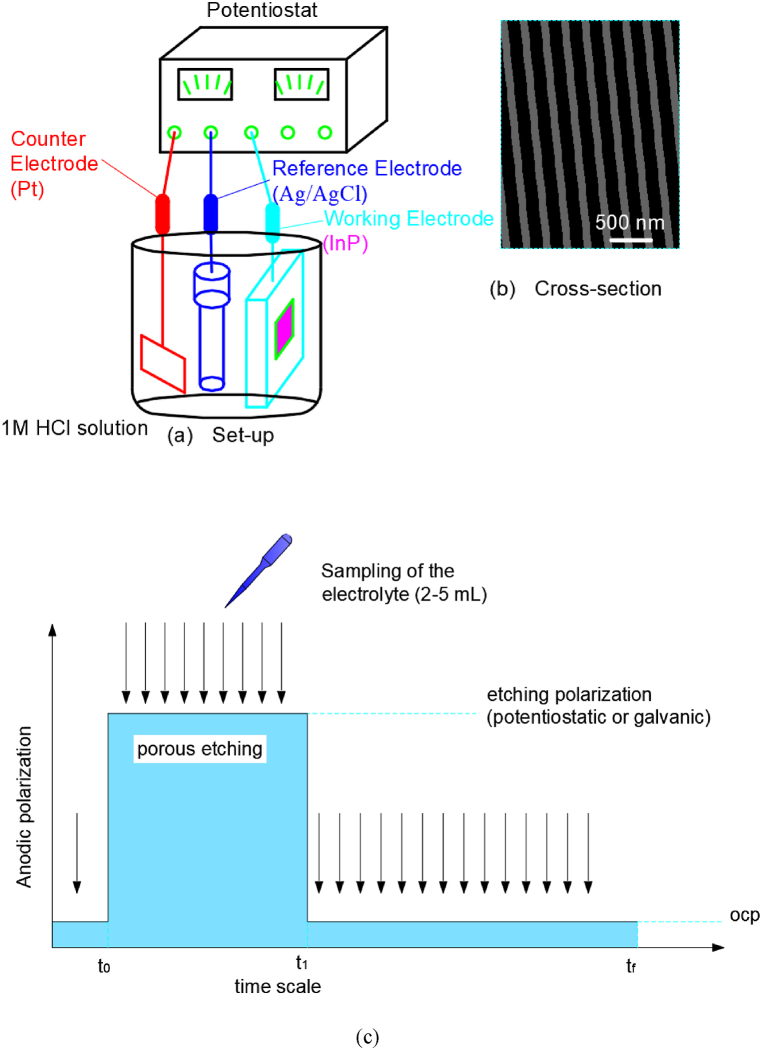
(A) Set-up for electrochemical etching of InP wafer; (b) Cross-section view; (c) Schematic diagram of the strategy used for analyzing the amount of emitted In^3+^. The black arrows represents the sampling cases.

Santinacci Lionel et al. [49] assembled porous layers on the surfaces of a *n*-InP slice in a HCl solution stirred by nitrogen gas. The whole experimental set-up was illustrated in Fig. 8(a). Indium phosphide wafers doped with Sn with 100 crystals were cut into 16 square millimeters and then rinsed with methanol. Hole etching was performed in a light-assisted three-electrode device consisting of the InP working electrode, a Pt line counter electrode and an Ag/AgCl reference electrode. Electrochemical etching was carried out under two different voltage and current conditions by a PAR 173 potentiostat.

The detection limit of indium by atomic absorption spectrometry was several hundred times more sensitive than that of phosphorus. The amount of dissolved indium phosphide could be deduced from the content of In^3+^. The model of atomic absorption spectrometer used here was Thermo Scientific M6. And the resulting morphology of the pit arrays in Fig. 8(b and c) shows that the pit width is 100–300 nm and the pit depth is 2–38 μm.

### 2.9 Electrochemical etching method 9

**Fig. 9:**
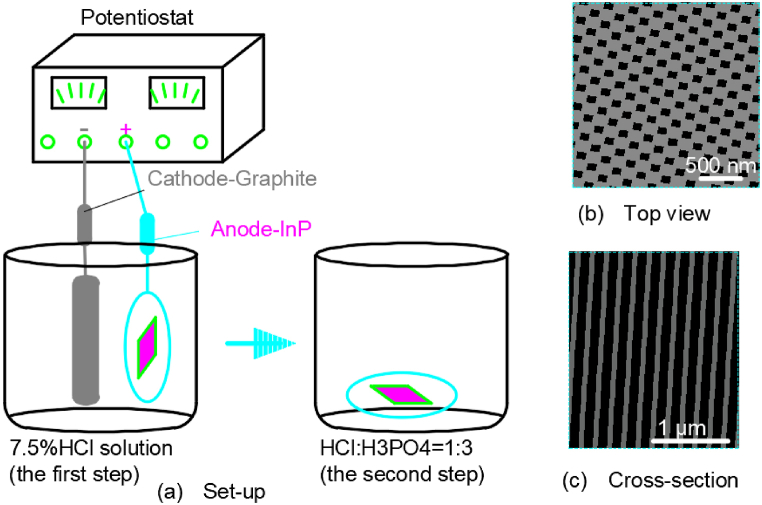
Configuration of a two-step etching approach for fabrication nanopore arrays on Indium Phosphide surface.

Zeng A S et al. [46] fabricated the porous InP layer with an etching pore density of about 5 × 10^4^ cm^−2^ by an anodizing electrochemical method in a 7.5% HCl solution. The whole experimental set-up was illustrated in Fig. 9(a). Before the experiment, indium phosphide wafers doped with Sn were rinsed with acetone and deionized water, respectively, and then dried with nitrogen. The indium phosphide wafer was pasted to the center of the copper sheet with conductive silver glue, and the exposed part of the copper sheet was coated with insulating paint. Indium phosphide chips served as the anode and graphite as the cathode in a Teflon tank. The wafer was polished with an oxalic acid solution under a voltage of 26 V to smooth the surface of the wafer. The wafer was then anodized in a dilute hydrochloric acid solution under a voltage of 7 V. Subsequently, the sample was placed in a mixture of pure hydrochloric and pure phosphoric acid (V_HCl_: V_H3PO4_ = 1 : 3) for 6 min at 20 °C to remove the irregular surface top layer. After ultrasonic cleaning in deionized water for 3 min, a uniform array of square holes on the surface of the indium phosphide wafer was formed. And the resulting morphology of the pit arrays in Fig. 9(b and c) shows that the pit width is ∼75 nm and the pit depth is 2–10 μm.

### 2.10 Electrochemical etching method 10

**Fig. 10:**
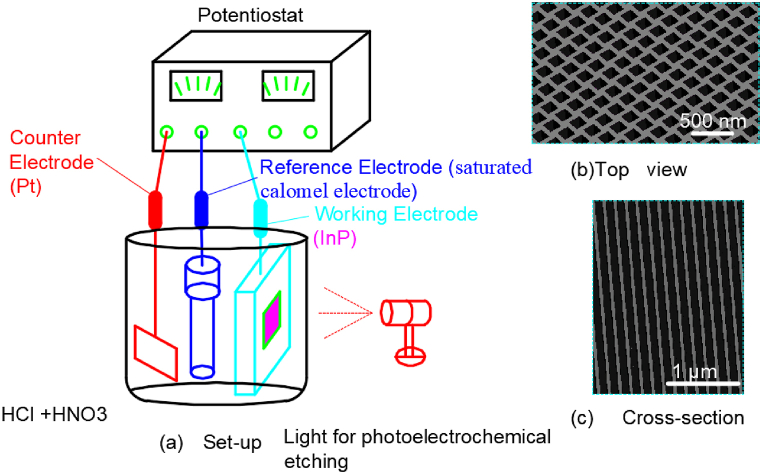
Set-up for electrochemical etching on the surfaces of InP wafer using light assistance with 0.01–1.0 mW in a HCl + HNO_3_ solution.

Liu A et al. [50] fabricated a porous array on the surface of indium phosphide wafer with 100 crystal direction and doped with tin. The whole experimental set-up was illustrated in Fig. 10(a). An InP working electrode, a Pt counterelectrode, and a reference saturated calomel electrode were used in the experiment. A mixture of hydrochloric acid and nitric acid were used as an electrolyte. And the resulting morphology of the pit arrays in Fig. 10(b and c) shows that the pit width is ∼180 nm and the pit depth is ∼17 μm.

### 2.11 Electrochemical etching method 11

**Fig. 11:**
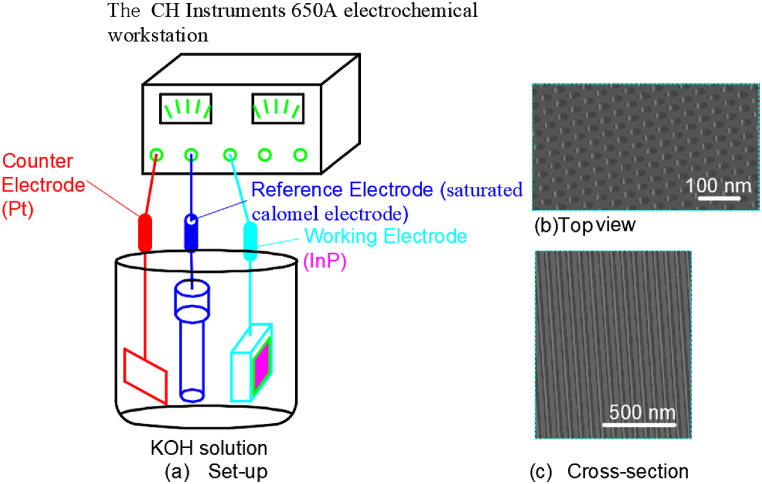
Set-up of anodization etching for an InP wafer in a 5 mol L^−1^ KOH solution.

Lynch R P et al. [45] etched indium phosphide chips in a potassium hydroxide solution at room temperature without light by a linear potential sweep at a certain speed. The whole experimental set-up was illustrated in Fig. 11(a). To make the working electrode, the wafer was cut into 5 mm × 5 mm square slices along the α and β directions, respectively. Ohmic contact was made by sputtering indium on the back of the wafer to increase electrical conductivity. The wafer was bonded to the surface of the copper sheet by silver paste, and the exposed part of the copper sheet was covered with insulating paint. The area of indium phosphide electrode was generally 0.2 square centimeters. Before entering the electrolytic cell, the indium phosphide working electrode was soaked in the paranha solution for 4 min and then rinsed with deionized water. There were three electrodes, namely, a platinum counter electrode, a saturated calomel electrode (SCE) and a working electrode. A CH Instruments Model 650 A Electrochemical Workstation was used for electrochemical parameter adjustment and data acquisition. And the resulting morphology of the pit arrays in Fig. 11(b and c) shows that the pit width is ∼20 nm and the pit depth is 0.5–1 μm.

### 2.12 Electrochemical etching method 12

**Fig. 12:**
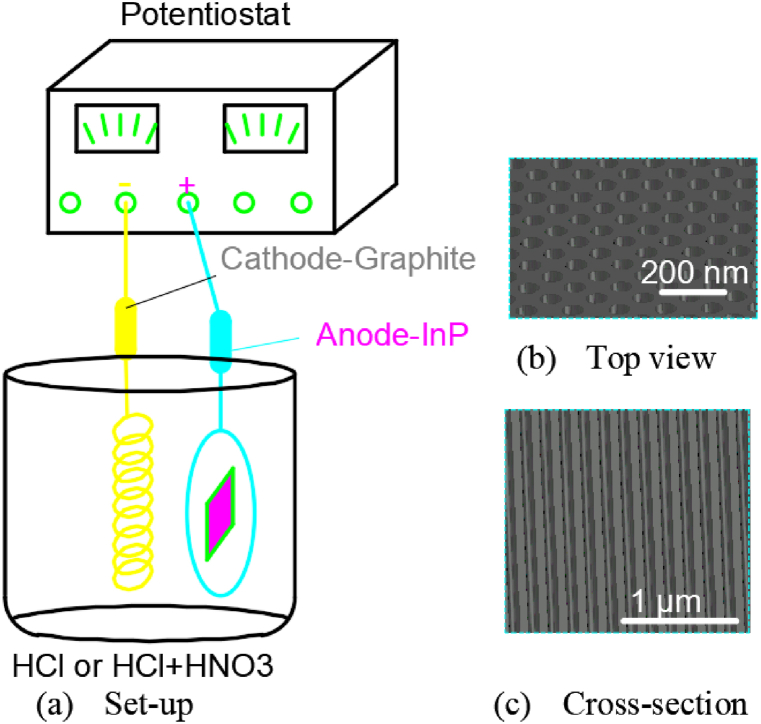
Electrolysis set-up for InP wafer.

Su G et al. [48] conducted an experiment in which an indium phosphide wafer coated with a patternized photoresist film was placed in a simple electrolysis device. The whole experimental set-up was illustrated in Fig. 12(a). Indium phosphide wafer with the crystal direction of 001 and the doped concentration of 10^18^ cm^−3^ was used as the anode. The wafers were cut into 5 mm × 5 mm and then ultrasonic cleaned with isopropyl alcohol or acetone. The wafer was then spin-coated with a 2 μm thick polymethyl methacrylate photoresist. After drying, the photoresist was covered with a glass template with a stripe pattern and exposed under UV light. The exposed photoresist was washed away with acetone.

An indium phosphide sample served as the anode, a ring of gold wire as the cathode. The voltage between the two electrodes was 2–10 V. The etching agent was a mixture of 1 mol L^−1^ hydrochloric acid and concentrated nitric acid (V_HCl_: V_HNO3_ = 50 : 1) or a hydrochloric acid solution with concentrations from 0.6 mol L^−1^ to 1.2 mol L^−1^. After electrochemical etching, the sample was first rinsed with deionized water and then washed with acetone to remove the sensitizer. And the resulting morphology of the pit arrays in Fig. 12(b and c) shows that the pit width is ∼64 nm and the pit depth is 1–8 μm.

## 3 Conclusions

In order to apply this review for a reference guide, a comparative analysis of the above methods is conducted in Table 1.

**Table 1:** The advantages and limitations of each electrochemical method.

No	Method name	Doping type	Electrolyte	Morphology	Comments	Suggestions	Ref.
1	Method 1	n	KOH	Pore width is 10–40 nm and pore depth is 0.8–2.8 μm	The pore diameter is smaller, the pore depth is shallower		52
2	Method 2	n	HCl	Pit width is 100–300 nm and pit depth is 0–150 nm	The pore diameter is appropriate, the pore depth is too shallow		14
3	Method 3	n	HCl	Pit width is 110–350 nm and pit depth is 0–10 nm	The pore diameter is appropriate, the pore depth is too shallow		15
4	Method 4	n	HCl/HCl + H_2_PtCl_6_/HCl + HNO_3_	Pit width is 50–180 nm and pit depth is 1–23 μm	The pore diameter is appropriate, the pore depth is also proper	Recommend	44
5	Method 5	n	HCl + H_2_PtCl_6_+NH_4_OH/HCl + HNO_3_	Pit width is 50–210 nm and pit depth is 2–80 μm	The pore diameter is appropriate, the pore depth is also proper	Recommend	40
6	Method 6	n	HCl	Pit width is 100–200 nm and pit depth is 2–15 μm	The pore diameter is appropriate, the pore depth is too shallow		5
7	Method 7	n	HCl	Pit width is 100–400 nm and pit depth is 5–50 μm	The pore diameter is appropriate, the pore depth is also proper	Recommend	11
8	Method 8	n	HCl	Pit width is 100–300 nm and pit depth is 2–38 μm	The pore diameter is appropriate, the pore depth is also proper	Recommend	49
9	Method 9	n	HCl	Pit width is ∼75 nm and pit depth is 2–10 μm	The pore diameter is smaller, the pore depth is shallower		46
10	Method 10	n	HCl + HNO_3_	Pit width is ∼180 nm and pit depth is ∼17 μm	The pore diameter is appropriate, the pore depth is also proper	Recommend	50
11	Method 11	n	KOH	Pit width is ∼20 nm and pit depth is 0.5–1 μm	The pore diameter is smaller, the pore depth is shallower		45
12	Method 12	n	HCl + HNO_3_	Pit width is ∼64 nm and pit depth is 1–8 μm	The pore diameter is smaller, the pore depth is shallower		48

It can be seen that the pores with bigger diameter and deeper depth were fabricated on the surfaces of InP wafers by method 4, 5, 7, 8, 10. The volume inside the pore is bigger with longer pit diameter and deeper pit depth, which can accommodate more chemical polymers. Therefore, it is more helpful to graft chemical polymers with various functional group such as acyl group, carboxyl group etc, which will be used to bond many biomolecules with an amino group for clinical tests.

## Author contribution statement

All authors listed have significantly contributed to the development and the writing of this article.

## Data availability statement

Data included in article/supplementary material/referenced in article.

## Declaration of competing interest

The authors declare that they have no known competing financial interests or personal relationships that could have appeared to influence the work reported in this paper.
